# Elucidating the Etiologies of 18F-fluorodeoxyglucose-Avid Mediastinal Lymph Nodes Among Cancer Patients in a Tuberculosis-Endemic Region Using Endobronchial Ultrasound

**DOI:** 10.7759/cureus.19339

**Published:** 2021-11-07

**Authors:** Usman Khalid, Muhammad J Akram, Muhammad Abu Bakar, Faheem M Butt, Mohammad B Ashraf

**Affiliations:** 1 Internal Medicine, Shaukat Khanum Memorial Cancer Hospital and Research Centre, Lahore, PAK; 2 Cancer Registry, Shaukat Khanum Memorial Cancer Hospital and Research Centre, Lahore, PAK

**Keywords:** mediastinal lymph node, cancer, tb, 18f-fdg, pet, ebus

## Abstract

Background

Non-malignant conditions, including infections (such as tuberculosis [TB]), can mimic malignancy with regards to their uptake of 18F-fluorodeoxyglucose (^18^F-FDG) tracer utilized for positron emission tomography-computed tomography (PET-CT) scan, as part of the diagnostic and staging workup of cancer patients. This poses a diagnostic challenge, for which tissue sampling is decisive. In this study, we aimed to determine the underlying etiologies of ^18^F-FDG-avid mediastinal lymph nodes among cancer patients in a TB-endemic demographic using endobronchial ultrasound-guided transbronchial needle aspiration (EBUS-TBNA) and the respective sensitivity and specificity of PET-CT and EBUS in diagnosing malignancy.

Methodology

In this retrospective cross-sectional study, we analyzed the data of all cancer patients with ^18^F-FDG-avid mediastinal lymphadenopathy on diagnostic PET imaging, who later underwent EBUS-TBNA between July 2013 and December 2018 at our center. Logistic regression analysis was used to determine the relative risk of lymph node characteristics with malignant TBNA cytology, based on which a risk stratification model was formulated.

Results

A total of 178 patients were included in this study, comprising predominantly males (60.7%). The primary malignancy was lung cancer in 33 (18.5%) patients, while 145 (81.5%) had non-lung cancer. A total of 214 ^18^F-FDG lymph nodes were sampled, out of which TBNA revealed malignant cytology in only 44 (20.6%). The final diagnosis was malignancy, TB, and sarcoidosis in 42 (23.6%), 16 (9%), and 12 (6.7%) patients, respectively. Among the remaining, 98 (55%) patients were determined to have only reactive lymphadenopathy, of which 24 (24.5%) had nodal anthracosis, while TBNA was inadequate for the diagnosis in 10 (5.6%) patients. An increased risk of malignancy was associated with the size of lymph node [odds ratio (OR): 1.58 (confidence interval (CI): 1.19, 2.11; p = 0.001], the standard uptake value (SUV) of the lymph node on PET-CT [OR: 1.30 (CI: 1.15, 1.45); p = 0.001], and with primary lung malignancy [OR: 4.44 (CI: 1.96, 10.06); p = 0.001]. At an SUV cut-off value of 6.0, PET-CT had the sensitivity, specificity, positive predictive value, and negative predictive value of 73%, 70%, 49.3%, and 91.8%, respectively, for diagnosing malignancy, while the same for EBUS was estimated to be 93.3%, 100%, 100%, and 97%, respectively.

Conclusions

In addition to TB, benign etiologies including nodal anthracosis and sarcoidosis predominate as causes of ^18^F-FDG-avid mediastinal lymphadenopathy in cancer patients of a TB-endemic demographic. The predictable risk of malignancy on PET imaging increases with nodal size, SUV, and lung primary malignancy; however, EBUS clearly demonstrates a higher sensitivity.

## Introduction

Positron emission tomography-computed tomography (PET-CT) plays a central role in the diagnostic imaging, treatment planning, and prognostication of cancer patients, including those with mediastinal lymphadenopathy [1-4]. However, the differential diagnosis of mediastinal lymphadenopathy can be vast, ranging from benign etiologies (including infections such as tuberculosis [TB]) to the more dreaded metastatic disease [5]. Consequently, the estimated sensitivity and specificity of PET-CT in determining malignancy is 70-80% and 59.8-71%, respectively [6,7]. Therefore, consensus guidelines unanimously recommend tissue sampling as histopathological evidence to establish a definitive diagnosis [8,9]. Previously, invasive surgical procedures including mediastinoscopy were regarded as the gold standard technique for lymph node sampling; however, this paradigm has convincingly shifted in favor of minimally invasive approaches, such as endobronchial ultrasound (EBUS), supported by an ever-increasing amount of evidence.

The diagnostic sensitivity and specificity of EBUS-guided transbronchial needle aspiration (EBUS-TBNA) for etiological diagnosis of mediastinal lymphadenopathy have been reported to be as high as 92% and 100%, respectively [5-7]. EBUS provides better access to hilar lymph nodes under real-time ultrasound guidance, with no major complications or procedure-related morbidity compared to invasive surgical techniques, such as mediastinoscopy [8-10].

However, tissue sampling has not superseded the necessity of PET-CT for most cancer patients [11]. The exact cut-off of the standard uptake value (SUV) of lymph nodes on PET-CT for determining the risk of malignancy has varied markedly in previously reported studies [12-14]. Making matters more complicated, TB and other granulomatous diseases (mainly sarcoidosis) mimic malignant tissues in terms of 18F-fluorodeoxyglucose (^18^F-FDG) avidity, thus further reducing the sensitivity and specificity of PET-CT [14]. The risk stratification for malignancy appears to be improved if other characteristics such as nodal size and SUV of primary malignant mass are considered simultaneously [13]. However, such an approach has rarely been replicated in TB-endemic demographics, where TB is an important cause of mediastinal lymphadenopathy mimicking malignancy [15-17]. In general, there is a dearth of studies on cancer patients from such regions, highlighting the incidence and nodal characteristics of malignant versus non-malignant ^18^F-FDG-avid mediastinal lymphadenopathy utilizing EBUS-TBNA, thus restricting the understanding concerning the risk stratification of lymph nodes for malignancy based on PET-CT.

The objective of this study is to elucidate the varying etiologies of ^18^F-FDG-avid mediastinal lymph nodes among cancer patients in our TB-endemic region utilizing EBUS-TBNA as the diagnostic tool. The study aims to determine the risk of malignancy in patients with mediastinal lymphadenopathy based on characteristic differences (e.g., SUV, size, etc.) between malignant and non-malignant etiologies in such patients by correlating the cytopathological and microbiological findings of EBUS-TBNA. In addition, the study aims to go a step further in aiming to formulate a risk stratification model for estimating the risk of malignancy based on these characteristic differences. Such an understanding and risk stratification model would help stratify the risk of malignancy based on PET-CT in a similar demographic setting, especially in resource-limited areas.

## Materials and methods

This retrospective cross-sectional study was granted approval by the Institutional Review Board (IRB) of the hospital. We acquired the data of all cancer patients who had undergone EBUS-TBNA at Shaukat Khanum Memorial Cancer Hospital and Research Centre, Lahore, between July 2013 and December 2018 (five years), and shortlisted patients who had undergone diagnostic imaging with PET-CT prior to the procedure for the diagnostic staging of the malignancy. The relevant clinical, radiological, and pathological data were collected by a detailed review of the electronic medical records of the patients.

All patients had undergone PET-CT following the routine hospital protocol. Prior to PET imaging, an intravenous (IV) injection of ^18^F-FDG was administered to each patient via large-bore IV cannulation. To allow for even distribution and uptake of the radiotracer, patients were allowed to rest quietly for 85 minutes in a shielded room. Imaging was performed on an integrated 16-slice PET-CT scanner, with scanning from the base of the skull to the mid-thigh. Baseline serum creatinine and blood sugar levels before ^18^F-FDG injection were measured in all patients. The reported SUVs of the lymph nodes were maximum SUVs unless stated otherwise. Patients with FDG-avid mediastinal lymphadenopathy were referred to a pulmonologist via multidisciplinary board discussions or through hospital clinics.

EBUS-TBNA was performed in each patient under routine IV sedation with midazolam and fentanyl, along with topical lignocaine sprays (2% and 4% concentrations). A convex-probe Olympus scope model 26BW (Olympus, Tokyo, Japan) and 22-gauge fine-needle aspiration (FNA) needle were utilized for this purpose. All FNA specimens were evaluated by a rapid on-site evaluation (ROSE) team to determine the adequacy according to the established criteria [18]. FNA specimens were separated for culture and cell block preparation. Each specimen underwent cytopathology block preparation, followed by relevant immunostaining, as well as *Mycobacterium tuberculosis* Gene Xpert assay, routine culture, and TB culture. The diagnosis of malignancy was established by the presence of malignant cells on the FNA cell block, and these patients were assumed to be true positives for PET-CT and EBUS for the diagnosis of malignancy. TB was diagnosed by the presence of granulomatous inflammation on cytopathology along with suggestive clinical history or with positive microbiology results (TB culture and/or Gene Xpert results), as per the standard guidelines [19]. The diagnosis of sarcoidosis was established by the presence of granulomatous inflammation, suggestive history, and negative TB culture/Gene Xpert. Patients with reactive cytopathology and negative microbiological workup were diagnosed with reactive lymphadenopathy.

A multivariable regression model was used to determine the risk of malignancy associated with lymph node size, SUV, and site of malignancy. By plotting the receiver operating characteristics (ROC) curve, a cut-off SUV value with comparable sensitivity and specificity for the diagnosis of malignant lymph nodes on PET-CT was determined. All lymph nodes above this cut-off value, but with a final diagnosis of benign lymphadenopathy on TBNA, were considered as false positives for PET-CT. Subsequently, the positive and negative predictive values (PPV and NPV) for PET-CT for malignancy were calculated.

The medical records of all patients were followed for one year after EBUS for any follow-up imaging and/or additional investigations. Patients were considered as false negatives for EBUS-TBNA where surgical biopsy through video-assisted thoracoscopic surgery (VATS) or mediastinoscopy led to a change of diagnosis, while patients remaining stable and with no further change of diagnosis or treatment were considered true negatives for malignancy. The overall sensitivity and specificity for EBUS-TBNA were computed using this approach.

## Results

A total of 178 cancer patients with ^18^F-FDG-avid mediastinal lymph nodes on PET imaging underwent EBUS-TBNA within the defined period, including 108 (60.7%) male patients. The mean age of the patients was 52.4 ± 16 years. The total number of lymph nodes sampled in these patients was 214. Overall, the primary cancer diagnosis was a lung malignancy in 33 (18.5%) patients, while the remaining had other cancers, as shown in Table 1. Overall, the TBNA cytology was consistent with malignancy in 44 (23.6%) lymph nodes sampled, as shown in Table 1.

**Table 1:** The key demographic variables and cancer diagnosis of patients along with the stations of lymph nodes sampled shown as the number and percentage of total. TBNA: transbronchial needle aspiration

Variable	Number (%)
Total patients	178
Gender	
Male	108 (60.7%)
Female	70 (39.3%)
Mean age of patients (years ± standard deviation)	52 ± 16
Primary malignancy	
Lung cancer	33 (18.5%)
Squamous cell carcinoma	15 (8.4%)
Adenocarcinoma	13 (7.3%)
Small cell carcinoma	04 (2.2%)
Poorly differentiated carcinoma	01 (0.6%)
Other cancers	145 (81.5%)
Esophageal carcinoma	42 (23.6%)
Hodgkin’s lymphoma	28 (15.7%)
Non-Hodgkin’s lymphoma	15 (8.4%)
Gastric carcinoma	15 (8.4%)
Colorectal carcinoma	09 (10.7%)
Gastroesophageal junction carcinoma	06 (3.4%)
Renal cell carcinoma	06 (3.4%)
Cervical/vaginal carcinoma	05 (2.8%)
Breast carcinoma	03 (1.7%)
Papillary thyroid carcinoma	03 (1.7%)
Ovarian carcinoma	03 (1.7%)
Pancreatic adenocarcinoma	03 (1.7%)
Endometrial carcinoma	02 (1.1%)
Sarcoma	02 (1.1%)
Prostatic adenocarcinoma	01 (0.6%)
Nasopharyngeal carcinoma	01 (0.6%)
Total lymph nodes sampled	214
Station 7	85 (39.7%)
Station 4R	60 (28%)
Station 11R	19 (8.9%)
Station 10R	11 (5.1%)
Station 11L	16 (7.5%)
Station 2R	06 (2.8%)
Station 4L	07 (3.3%)
Station 2L	06 (2.8%)
Station 5	04 (1.9%)
TBNA cytology	
Malignant	44 (20.6%)
Benign or reactive	125 (58.4%)
Granulomatous	33 (15.4%)
Inadequate	12 (5.6%)

For the total lymph nodes sampled, the TBNA cytology was malignant in 44 (20.6%), benign or reactive in 125 (58.4%), and granulomatous in 33 (15.4%), while cytology was inadequate for cytological diagnosis in 12 (5.6%) patients. The final diagnosis was consistent with malignant lymphadenopathy in 42 (23.6%) patients, while the diagnosis of TB and sarcoidosis was established in 16 (9%) and 12 (6.7%) patients, respectively. In total, 98 (55.1%) patients with ^18^F-FDG-avid lymph nodes were determined to have benign lymphadenopathy on TBNA. TBNA was inadequate for cytological analysis in 10 (5.6%) patients; thus, the overall adequacy for EBUS-TBNA was 94.3%. Nodal anthracosis was found in 24 (24.5%) patients with benign diagnoses, while necrosis was reported in 6 (6.1%) such patients on the final cytopathology report.

The mean SUV of malignant lymph nodes was determined to be 8.4 ± 4.5, and the mean difference of SUV between malignant and non-malignant etiologies was statistically significant (p = 0.001). The mean size of mediastinal lymph nodes sampled was 1.65 ± 0.84 cm. The mean size of mediastinal lymph nodes was 2.41 ± 1.6 cm in patients diagnosed with malignancy, and this was significantly higher than non-malignant etiologies (p = 0.001), as shown in Table 2. The mean SUV of lymph nodes was higher in patients diagnosed with sarcoidosis versus TB (Table 2).

**Table 2:** The mean lymph node size and SUVs with respect to the final diagnosis of patients. *SD; **SUV. SUV: standard uptake value; SD: standard deviation

Variable	Malignant	Tuberculosis	Sarcoidosis	Reactive	Inadequate	P-value
Final diagnosis (out of 178 patients)	42 (23.6%)	16 (9%)	12 (6.7%)	98 (55%)	10 (5.6%)	-
Mean size of lymph nodes (cm ± SD*)	2.41 ± 1.64	2.06 ± 2.02	1.42 ± 0.32	1.53 ± 0.92	1.19 ± 0.61	0.001
Mean SUV** of lymph nodes ± SD*	8.4 ± 4.5	6.0 ± 3.1	6.4 ± 3.6	5.2 ± 2.3	4.6 ± 1.9	0.001

In 103 (57.9%) patients, the SUV of the primary malignant lesion was compared to that of the sampled mediastinal lymph node(s), and the percentage ratio of the SUV of the lymph node to the primary lesion was calculated. Of these patients, 30 (29%) had malignant TBNA cytology, with the mean lymph node to primary mass SUV ratio of 81.6 ± 37.4, while 73 (71%) had benign TBNA cytology, with the mean ratio of 64.85 ± 64.1; however, the difference in means between the two groups was not statistically significant (p = 0.2).

The percentage of malignant lymphadenopathy was higher among patients with pulmonary malignancies (47%) compared to other cancers (11.2%), and there was a statistically significant difference between the mean SUV of malignant and non-malignant lymph nodes among these groups (p = 0.000), as shown in Table 3.

**Table 3:** The mean difference of SUV of lymph nodes with malignant versus non-malignant cytology with respect to the primary origin of the malignancy. *SUV; **SD. SUV: standard uptake value; SD: standard deviation

	Lung cancer	Other cancers	P-value
Mean SUV* ± SD** (Malignant)	10 ± 3.9	8.75 ± 4.5	0.0001
Mean SUV* ± SD** (Non-malignant)	3.3 ± 1.14	5.4 ± 2.4	0.0001

**Figure 1:**
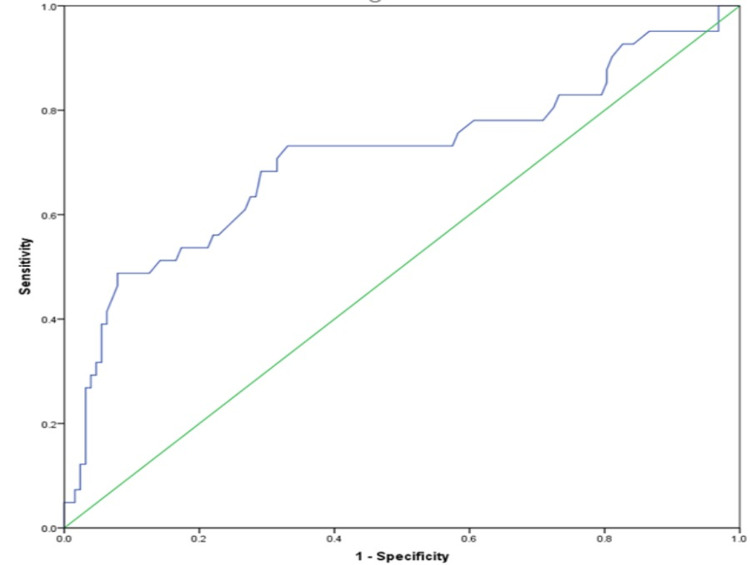
ROC curve showing AUC of 70% at an SUV cut-off of 6.0, with a sensitivity of 73% and specificity of 70%. ROC: receiver operating characteristics; AUC: area under the curve; SUV: standard uptake value

Using the ROC curve, an SUV cut-off of 6.0 was determined to have a sensitivity and specificity of 73% and 70%, respectively, for the diagnosis of malignancy (Figure 1).

At the SUV of 6.0, the PPV and NPV of PET-CT for diagnosing malignancy were estimated to be 49.4% (false positive: 45) and 91.8% (false negative: 14), respectively. All patients with a benign diagnosis underwent radiological and clinical surveillance. VATS diagnosed malignancy and TB in one patient each, who previously had benign TBNA cytology on EBUS. One patient had a diagnosis of malignancy on endoscopic ultrasound (EUS)-guided fine-needle aspiration (EUS-FNA) of the lymph node. These three patients were assumed to be false negative for EBUS, while the remaining 95 were considered true negatives. Based on this premise, the sensitivity, specificity, PPV, and NPV of EBUS-TBNA were estimated to be 93.3%, 100%, 100%, and 97%, respectively.

Using the multivariate regression analysis, lymph node size, SUV, and lung origin of primary cancer were determined to be risk factors for malignant lymphadenopathy, as shown in Table 4.

**Table 4:** Multivariate logistic regression model to determine the OR of the risk of malignant lymphadenopathy on PET-CT with respect to the size, SUV, and location of the malignancy. OR: odds ratio; CI: confidence interval; SD: standard deviation; SUV: standard uptake value; PET-CT: positron emission tomography-computed tomography

Variables	Unadjusted OR (CI)	P-value	Adjusted OR (CI)	P-value
Size of lymph node (mean ± SD)	1.58 (1.19, 2.11)	0.001	1.33 (1.00, 1.82)	0.07
SUV of lymph nodes (mean ± SD)	1.30 (1.15, 1.45)		1.26 (1.11, 1.42)	0.001
Location of malignancy				
Extrathoracic	Reference		Reference	
Thoracic (lung)	4.44 (1.96, 10.06)	0.001	4.50 (1.71, 11.80)	0.002

Based on these findings, a risk stratification tool was formulated for the prediction of malignancy in FDG-avid lymph nodes. This score was applied to all 178 patients in the study population to determine the appropriate cut-off for diagnosing malignant lymphadenopathy. The formulated score is shown in Table 5. The score showed a sensitivity of 64.3% and a specificity of 73.2%, with PPV and NPV of 44.3% and 86.1%, respectively, at a cut-off score of 4. When the cut-off score was raised to 6, the specificity and PPV increased to 100%, and the sensitivity and NPV were 33% and 79%, respectively. The score table and results are shown in Table 5 and Table 6, respectively.

**Table 5:** Scoring model for the risk stratification of lymph nodes for estimating the risk of malignancy based on high-risk characteristics. SUV: standard uptake value

Variable	Score		
	0	1	2
Size of lymph node (cm)	<1	1-2	>2
SUV of lymph node	<5.0	5-6.0	>6
Location of primary tumor	-	Other cancers	Lung cancers

**Table 6:** Results of the scoring of patients included in the study as per the suggested scoring model with respect to the final etiological diagnosis of malignancy, TB, sarcoidosis, and benign lymphadenopathy. TB: tuberculosis

Total score (maximum 6)	Malignancy (n = 42)	TB (n = 16)	Sarcoidosis (n = 12)	Benign (n = 99)	Inadequate (n = 10)
1	2 (4.8%)	2 (12.5%)	0	17 (17.2%)	4 (40%)
2	9 (21.4%)	7 (43.8%)	7 (58.3%)	36 (36.4%)	4 (40%)
3	4 (9.5%)	2 (12.5%)	1 (8.3%)	21 (21.2%)	0
4	13 (31%)	5 (31.3%)	4 (33.3%)	25 (25.3%)	2 (20%)
6	14 (33.3%)	0	0	0	0

## Discussion

Despite its vital role in cancer imaging, PET-CT has been found to have a lower sensitivity, particularly in TB-endemic regions, to correctly distinguish between malignant and non-malignant diseases [14,20]. This study determines the etiologies of ^18^F-FDG-avid mediastinal lymph nodes in cancer patients with mediastinal lymphadenopathy belonging to a TB-endemic demographic. Our study estimated a sensitivity of 93% and a specificity of 100% for EBUS-TBNA for the diagnosis of malignant mediastinal lymphadenopathy, which compares well with previously reported data [21-23].

Similar previous studies have attempted to ascribe an SUV cut-off values for malignant mediastinal lymphadenopathy, while others have determined nodal size, SUV of primary malignant mass, and sonographic features of lymph nodes on EBUS to be associated with an increased risk of malignancy. In a study by Lilo et al., 42% of patients with ^18^F-FDG-avid mediastinal lymphadenopathy had a malignant disease, with an SUV cut-off value of 4.5 associated with a high risk of malignancy. However, there was no statistically significant difference in the size of lymph nodes between malignant and non-malignant lymph nodes, as well as no mean difference of SUV of malignant nodes in patients with lung versus other non-lung cancers [12]. In two studies, the cut-off of SUVs was suggested to be approximately 5.2 [22,23]. Evison et al. determined the SUV cut-off value of 4.5, nodal heterogenous echogenicity on EBUS, and lymph node to primary mass SUV ratio of >60% to be significant factors associated with an increased risk of malignancy [13]. Kumar et al. reported the results from a population with high TB endemicity and postulated an ideal SUV cut-off to be approximately 6.2. In addition, they reported a statistically significant difference in SUV between benign and malignant conditions and no difference among benign conditions such as TB and sarcoidosis or the size of lymph nodes among malignant or non-malignant etiologies [14].

In our setting, metastatic lymphadenopathy was diagnosed in only 23.6% of patients, and the cut-off SUV value (6.0) for malignancy was higher than previously reported in non-TB endemic settings, with a resultant lower sensitivity (73%), specificity (70%), PPV (49.3%), and NPV (91.8%) of PET-CT for the diagnosis of malignancy. In other words, in a TB-endemic demographic, nodal SUV alone has a low PPV for malignancy. In our study, there was a statistically significant difference in the mean SUV of malignant and non-malignant lymph nodes. Additionally, the mean SUV was higher in patients ultimately diagnosed with sarcoidosis compared to those with TB, while the mean lymph node size was lower in the former. Nodal anthracosis was detected in 24.4% of patients with benign lymphadenopathy. Nodal anthracosis has previously been acknowledged to be an important cause of ^18^F-FDG-avid lymphadenopathy [24]; however, the incidence in patients with a background malignancy has not been reported.

Unlike previous studies, there was a significantly higher incidence of malignant lymphadenopathy (47%) in patients with primary lung malignancy. This study determines that at a given SUV, the risk of malignancy is nearly 4.5 times higher in mediastinal lymph nodes in a patient with lung cancer versus non-lung cancers, along with a higher incidence of granulomatous diseases in the latter. This percentage of metastatic lymphadenopathy was previously described to be higher in patients with extrathoracic cancers (68%) belonging to the non-TB-endemic region [25]. However, similar to our results, this percentage was determined to be lower (31%) in a study in a TB-endemic region [26]. In either of these studies, the incidence of extra-thoracic cancers was not compared to lung cancers.

The ratio of SUV of primary malignant mass to SUV of lymph nodes has been estimated to be significantly higher in patients with metastatic versus benign lymphadenopathy [13,27,28]. In contrast, in our study, this ratio was not statistically significant in terms of the mean difference between patients ultimately diagnosed with malignant versus benign lymphadenopathy by TBNA. One reason could be the lower number of comparable patients with malignant lymphadenopathy (23.6% versus up to 78% in previous studies).

While the surgical biopsy is often regarded as the gold standard, the risk of a false-negative diagnosis on EBUS-TBNA has been determined to be low. In a previous study, the results of EBUS-TBNA were compared to surgical lymph node biopsy in all patients with a benign (negative) TBNA cytopathology, concluding that just 4.9% of patients were false negative for malignancy [29]. This percentage is too low to justify invasive biopsy in all patients with negative results, especially in cancer patients with a poor performance status [15]. In our study, a multifactorial risk stratification score was formulated, utilizing nodal size and primary origin of malignancy, in addition to nodal SUV. The suggested score improves the PPV for malignancy to up to 100%. The score does not supersede the importance of tissue sampling, given the lower sensitivity; however, it can help identify possible false-negative patients who can then either receive close follow-up or immediate surgery. Evison et al. proposed a risk stratification model to assess false-negative lymph nodes on EBUS-TBNA based on lymph node SUV, lymph node/mass SUV ratio, and sonographic echotexture of the lymph node [13]. However, in our study population, the lymph node/mass SUV ratio was not statistically significant, as mentioned earlier. Such a risk stratification tool is novel for a TB-endemic setting and is beneficial in resource-limited settings.

We wish to acknowledge certain limitations of this study. First, because the study centers on ^18^F-FDG-avid (SUV > 2.5) lymph nodes, it does not determine the risk of malignancy in those labeled as non-avid on PET-CT. In a previous study, this risk was reported to be up to 17.6% [30]. Nodal echotexture on EBUS was not commented upon, which was due to the lack of uniform reporting in all patients. Finally, the risk stratification model should undergo a prospective review to estimate its true sensitivity and specificity, which was not done within the scope of this study. However, because cancer patients usually have limitations of performance status [15], surgical biopsy in each patient to exclude a false negative is not justifiable and betrays the intent of a minimally invasive diagnostic approach via EBUS. Despite these limitations, the study represents robust findings and suggests a possible way of stratifying the risk of malignancy in ^18^F-FDG-avid lymph nodes in patients within a TB-endemic demographic.

## Conclusions

This study reveals that the etiology of ^18^F-FDG-avid mediastinal lymphadenopathy in cancer patients is predominantly benign, and etiologies such as TB, sarcoidosis, and nodal anthracosis account for a significant number of such patients. The cut-off SUV for estimating the risk of malignancy on PET-CT is higher in TB-endemic demographics, with a poorer sensitivity and specificity compared to non-TB-endemic settings. There appears to be a significantly higher risk of metastatic lymphadenopathy in ^18^F-FDG-avid mediastinal lymph nodes of a lung primary cancer compared to extrathoracic cancers, as well as with lymph node size of >2 cm and SUV of >6.0. 
